# Factors Influencing Early Health Facility Contact and Low Default Rate among New Sputum Smear Positive Tuberculosis Patients, India

**DOI:** 10.1155/2014/132047

**Published:** 2014-03-06

**Authors:** Ashok Kumar Bhardwaj, Surender Kashyap, Pradeep Bansal, Dinesh Kumar, Sunil Kumar Raina, Vishav Chander, Sushant Sharma

**Affiliations:** ^1^Department of Community Medicine, Dr. Rajendra Prasad Government Medical College, Kangra, Himachal Pradesh 176001, India; ^2^Kalpana Chawla Government Medical College, Karnal, Haryana, India

## Abstract

Early case identification and prompt treatment of new sputum smear positive case are important to reduce the spread of tuberculosis (TB). Present study was planned to study the associated factors for duration to contact the health facility since appearance of symptoms and treatment default. *Methodology*. It was prospective cohort study of TB patients already registered for treatment in randomly selected TB units (TUs) in Himachal Pradesh, India. Relative risk (RR) was calculated as risk estimate to find out the explanatory variables for early contact and default. *Results*. Total 1607 patients were recruited and 25 (1.5%) defaulted treatment. Patients from nuclear family (aRR: 1.37; 1.09–1.73), ashamed of TB (aRR: 1.32; 1.03–1.70), wishing to disclose disease status (aRR: 1.79; 1.43–2.24), but aware of curable nature (aRR: 1.67; 1.17–2.39) and preventable (aRR: 1.35; 1.07–1.70) nature of disease, contacted health facility early since appearance of symptoms. *Conclusion.* Better awareness and less misconceptions about disease influences the early contact of health facility and low default rate in North India.

## 1. Introduction

One-third of the world population is infected with *Mycobacterium tuberculosis* causing disease in about 10 million individuals and resulting in 1.3 million deaths per year. Asia and Africa regions together share 85.0% of the global disease burden [1]. India notified total 1.3 million cases including 0.62 million sputum smear positive cases [2]. Evidence from India showed that tuberculosis (TB) contributes about 30% of deaths due to communicable disease and 7% of total deaths [2]. Based upon World Health Organization (WHO) recommendations, the Government of India implemented Directly Observed Treatment Short Course (DOTS) strategy under Revised National Tuberculosis Program (RNTCP) [2]. Since then, treatment success rate among sputum smear positive patients improved from 25% in the year 1985 to about 90% by the year 2011 [2] as compared to the global treatment success rate of 84% [1]. Early case detection and treatment is a public health principle for disease control. Under RNTCP, an awareness campaign for signs and symptoms of TB demands an early contact of health facility for diagnosis. Contact of patient to health facility for treatment compliance depended on the program and patient related factors [3–5]. A lot has been done for disease awareness under program; the community behavior becomes supportive toward patient. However, evidence also showed that still stigma associated with signs and symptoms of TB, availability of diagnostic, and treatment services under RNTCP. Patients tend to hesitate and choose not to disclose their disease status to their family/friends out of fear of being socially marginalized [3–6]. The nature and degree of influence of such factors on health facility contact and so compliance for DOTS have not been reported as often. Therefore, the present study was planned to understand the role of factors in contacting a health facility since the appearance of symptoms and treatment default among patients on DOTS under RNTCP, Himachal Pradesh, India.

## 2. Methodology

It was a prospective cohort study of two years' (2008-09) duration conducted at randomly selected tuberculosis units (TUs) in the state of Himachal Pradesh, India. As per the census of 2011, the state has population of about 7 million spread over 55, 670 km^2^ area residing in 12 districts. Sample size of 1537 was calculated with default rate of 4.0% (based upon the selected TUs for study) at 5% significance level and 80% of study power. Sample size was revised to 1691 with 10% nonresponse rate. Interview was done to collect the data by the trained project staff using pretested and structured questionnaire.

Patients were getting DOTS for the period of six months from the DOTS providers as per the RNTCP. All new smear positive (NSP) TB cases with more than 15 years of age already on DOTS from selected TUs of study area were recruited. Once recruited every patient was contacted three times: first, as soon as they started with DOTS, second, at the end of intensive phase of DOTS, and, third, one week before completion of DOTS (i.e., at the time of administration of last dose of DOTS). A patient was considered to be a defaulter if he/she interrupted the treatment for two consecutive months or more. All the defaulted patients were contacted to assess the reason for default and were motivated to continue the DOTS. Based on review, studies differentially had used the definition for “delay in health facility contact” and no such clear definition is being used under RNTCP. Therefore, a cutoff of 30 days for deciding the early facility contact was considered after calculation of central tendency (median) of duration (days) of health facility contact since the appearance of symptoms.

RNTCP is providing DOTS to the patients through total 41 TUs in the state. Total 20 TUs were selected based upon population proportion to size (PPS) using the cluster sampling technique. Retrospective information was collected from patients already on DOTS who were interviewed about their place of residence, type of family, socioeconomic class, appearance of symptoms, time of contact to a health facility, substance abuse, disease knowledge, and misconceptions. Information about the health system related factors like distance of health facility from the place of residence and waiting time at centre and exact treatment duration explained by the DOTS provider was also asked from the patient. Prospective information in next two subsequent visits was also collected for sputum conversion, drug side effects, and family and society response towards the patient. If defaulted patient did not respond to the DOTS then patient was contacted prospectively at the place of residence by the project staff to assess the reasons for default. Data was collected on structured questionnaire and was translated into local language and back to English before the data collection. Before recruiting the patient an informed consent was taken in the first visit explaining that there would be further collection of information in the next two consecutive visits. A prior ethical clearance from institutional ethics committee was also obtained.

Data was entered and analyzed using Epi Info software version 3.3.2 for Windows. Effect size in terms of relative risk (RR) with 95% confidence interval (CI) was also calculated. The continuous variables were recoded into categorical variable and the reference category was selected and was compared with the rest of categories. Multivariate analysis using bimodal logistic regression was carried out for control of confounding and adjusted effect size (aRR) was computed. Multivariate analysis was not carried out for defaulters and nondefaulter patients due to less (25) number of treatment defaulters.

## 3. Results

Total 1607 patients were enrolled from all the randomly selected TUs and followed up in the study. Patients were largely from rural (80.0%) area, male (67.9%), and of 15–44 years (62.3%) of age group. Almost half of the patients had joint family of middle socioeconomic class (65.5%) with more than three members (86.5%) in the family. Majority reported alcohol (56.9%) consumption and smoking (54.4%) at the time of interview. It was observed that only 25 (1.5%) patients defaulted (1.5%) DOTS. Comparatively, the number of defaulted patients was small to look for statistically significant difference, but most of patients (76.0%) thought that TB and its treatment affect their work performance, 56.0% hid their disease status from others, and 52.0% did not disclose their disease status to their family members. Despite this, 96.0% of defaulted patients were aware of the curable nature of disease and 84.0% knew about the duration of available treatment (data not shown).

Since appearance of symptoms, patients were put on DOTS after median (mean: 49 days; mode: 33 days) duration of 33 days. Treatment was started after more than 30 days among 55.6% of patients. Once patient contacted the health facility, treatment was started within 2.4 day. Most of the patients (62.1%) first contacted government health facility for care. Almost all (95.5%) patients were on category I treatment of DOTS.

Significantly early (within 30 day) contact to health facility was observed among patients of upper socioeconomic status (RR: 1.80; CI: 1.72–1.88), nuclear family (RR: 1.27; CI: 1.04–1.55) with family size of 3–5 (RR: 1.22; CI: 1.00–1.49), and of rural area (RR: 1.55; CI: 1.20–2.00). Early contact was observed less among patients of urban area (RR: 0.64; CI: 0.48–0.83) (Table 1). When analyzed for patient related factors, it was found that significantly more patients contacted health facility early, who felt ashamed about their disease status (RR: 1.41; CI: 1.15–1.74), but, ready to disclose their disease status (RR: 1.63; CI: 1.33–1.98), thought that treatment would be costly (RR: 2.08; CI: 1.40–3.09), and they knew disease is curable (RR: 1.96; CI: 2.43–2.69) and could be prevented by vaccine (RR: 1.29; CI: 1.05–1.59). Significantly fewer patients contacted early who wished to hide the disease from others (RR; 0.80; CI: 0.65–0.97). It was also observed that the exact treatment duration was explained by DOTS provider (RR: 1.96; CI: 1.26–3.04) to patients who contacted health facility early (Table 2).

Multivariate analysis showed that significantly more patients contacted health facility early and they were males (aRR: 1.55; CI: 1.18–2.04), from nuclear family (aRR: 1.37; CI: 1.09–1.73) (Table 1), and ashamed of their disease status (aRR: 1.32; CI: 1.03–1.70), wished to disclose disease status (aRR: 1.79; CI: 1.43–2.24), thought treatment would be costly (aRR: 2.45; CI: 1.59–3.78) but were aware of curable nature (aRR: 1.67; CI: 1.17–2.39) of disease and aware of available vaccine (aRR: 1.35; CI: 1.07–1.70) for prevention and health facility (aRR: 1.72; CI: 1.08–2.74) which was more than 60 minutes away from place of residence (Table 2). Fewer patients contacted the health facility early and they were from urban area (aRR: 0.69; CI: 0.51–0.93) (Table 1), with family size of 3–5 (aRR: 0.68; CI: 0.48–0.97), 6-7 (aRR: 0.60; CI: 0.41–0.89), wished to hide status from others (aOR: 0.69; CI: 0.51–0.93), and thought that disease would affect daily work (aRR: 0.73; CI: 0.56–0.97) performance. It was observed that the correct treatment duration was explained by the DOTS provider (aRR: 1.88; CI: 1.12–3.15) to patients who contacted health facility early (Table 2).

## 4. Discussion

With about 2 million deaths a year in the world and 7.0% of total deaths in India, TB is a prevalent public health problem [1, 2, 7]. In the last 50 years, reduction in disease burden in India had been observed as national TB control efforts were expanded to the entire county [7, 8]. Early diagnosis and treatment of the case to break the chain of transmission were vital for disease control. Usually patients did not report to health facility early due to perceived mild nature of symptoms, bad staff behavior, and patient dissatisfaction [9, 10]. Societal issues like expected problems related to social status, marriage, and adverse community behavior were major reasons for stigmatizing behavior of patient [11, 12]. These factors result in late case identification and result in disease transmission in the community. Present study showed that the patients who contacted health facility early were those who felt ashamed of the disease but ready to disclose disease status to others. Few patients contacted the health facility early because they wished to hide their disease status from others and thought that the disease would affect their daily work performance. Present study observed weak evidence for disease effect on work capacity, family relations, marriage, and female infertility (Table 2).

Prior disease knowledge and awareness influence the health care seeking behavior. It was observed that patients who were on treatment were aware of infectious nature of disease and its available treatment [13]. Present study also showed that patients who contacted health facility early were aware of the preventable and curable nature of disease. In present study, correct treatment duration was explained by the DOTS provider among patients who contacted the health facility early. Present study observed average duration of delay of 30 days which is low as compared to about 56 reported by other studies in an African country [14]. This difference amounts to perceived nature of symptoms and penetration of program in terms of availability and accessibility of management services in the country [3–6].

In India, treatment success rate approached 85.0% and met the set objective under RNTCP [15]. Poor treatment compliance could lead to high default rate and pose threat to multidrug resistant tuberculosis. However, present study had showed default rate of 1.5% similar to 1.1% as observed in North India [16]. Present study observed that defaulted patients still did not disclose their disease status to their family members and friends despite their correct knowledge about the duration of treatment and curable nature of disease. Smoking [7] and alcohol [17, 18] are known to increase the risk of mortality and high default rate among patients with substance abuse [12]. Present study observed insignificant but high prevalence of alcohol (76.0%) and smoking (12.0%). Against the reported (before the study was based upon routine health information system) default rate of 3.8% of selected TUs, present study observed low default which could be due to two prospective visits study design, in addition to the usual inbuilt patient monitoring under the program.

In the present study, disease awareness and complete information provided by the DOTS provider influenced early contact of health facility and low treatment default rate. High level of advertisement under RNTCP possibly had played a significant role in the study area. Low default rate could be attributed to study design itself as patient was approached by the study staff three times. It poses a limitation of study as it could not the controlled in study design, but it shows that additional visits to contact patient could reduce the treatment default rate. RNTCP has strengthened itself to achieve high case detection and cure rate. It will substantiate the successful efforts in the state to combat TB as one of the top ten leading killers.

## Figures and Tables

**Table 1:** Demographic profile among TB patients with default and delay treatment, Himachal Pradesh, India, 2008-09.

Characteristics	Delay (<30 day)	Delay (>30 day)	Risk estimate	Risk estimate
	(713)	(894)	(Unadjusted)	(Adjusted)
	*N* (%)	*N* (%)	RR (95% CI)	aRR (95% CI)
Age group (years)				
15–24	191 (27.2)	208 (23.5)	1.23 (0.98–1.50)	Ref
25–34	156 (22.2)	172 (19.4)	1.17 (0.92–1.48)	1.05 (0.75–1.47)
35–44	111 (15.8)	152 (17.2)	0.90 (0.68–1.17)	1.39 (0.93–2.07)
45–54	92 (13.1)	137 (15.5)	0.81 (0.61–1.08)	1.46 (0.97–2.21)
55 and above	153 (21.8)	217 (24.5)	0.85 (0.67–1.07)	1.26 (0.87–1.82)
Sex				
Male	501 (70.3)	590 (66.0)	1.21 (0.98–1.50)	1.55 (1.18–2.04)
Status				
Married	479 (67.2)	622 (69.6)	0.89 (0.72–1.10)	1.05 (0.78–1.40)
Socioeconomic status				
Upper	0 (0.0)	07 (00.8)	**1.80** (1.72–1.88)*	—
Upper middle	167 (23.4)	174 (19.5)	1.26 (0.60–1.99)	0.47 (0.44–1.54)
Lower middle	310 (43.5)	402 (45.0)	0.91 (0.77–1.14)	0.54 (0.16–1.75)
Upper lower	231 (32.4)	302 (33.8)	0.93 (0.76–1.15)	0.55 (0.17–1.76)
Lower	5 (0.7)	009 (01.0)	0.69 (0.23–2.08)	Ref
Religion				
Hindus	686 (96.2)	855 (95.6)	1.15 (0.70–1.91)	1.06 (0.62–1.82)
Family				
Nuclear	361 (50.6)	399 (44.6)	**1.27** (1.04–1.55)*	**1.37** (1.09–1.73)*
Family size				
<3	75 (10.5)	141 (15.8)	**0.62** (0.46–0.84)*	Ref
>3	333 (46.7)	372 (41.6)	**1.22** (1.00–1.49)*	**0.68** (0.48–0.97)*
Place of residence				
Rural	597 (83.8)	688 (77.0)	**1.55** (1.20–2.00)*	Ref
Urban	98 (13.8)	180 (20.1)	**0.63** (0.48–0.82)*	**0.69** (0.51–0.93)*
Alcohol				
Yes	416 (58.3)	499 (55.8)	1.10 (0.90–1.35)	1.21 (0.93–1.59)
Smoking				
Yes	72 (10.1)	094 (10.5)	0.95 (0.69–1.32)	0.91 (0.63–1.30)

*Statistically significant.

**Table 2:** Disease awareness among TB patients with default and delay treatment, Himachal Pradesh, India, 2008-09.

Characteristics	Delay (<30 day)	Delay (>30 day)	Risk estimate	Risk estimate
	(713)	(894)	(Unadjusted)	(Adjusted)
	*N* (%)	*N* (%)	RR (95% CI)	aRR (95% CI)
Stigma				
Ashamed	271 (38.0)	270 (30.2)	**1.41** (1.15–1.74)*	**1.32** (1.03–1.70)*
Hide from others	402 (56.4)	552 (61.7)	**0.80** (0.65–0.97)*	**0.70** (0.55–0.88)*
Disclose to others	384 (53.9)	373 (41.7)	**1.63** (1.33–1.98)*	**1.79** (1.43–2.24)*
Hereditary	300 (42.1)	362 (40.5)	1.07 (0.87–1.31)	1.03 (0.81–1.31)
Prefer to be isolated	181 (25.4)	212 (23.7)	1.09 (0.87–1.37)	1.08 (0.83–1.39)
Costly treatment	068 (09.5)	043 (04.8)	**2.08** (1.40–3.09)*	**2.45** (1.59–3.78)*
Effect				
Work	502 (70.4)	661 (73.9)	0.83 (0.67–1.04)	0.73 (0.56–0.97)
Marriage	218 (30.6)	270 (30.2)	1.01 (0.82–1.26)	0.99 (0.77–1.28)
Responsibility	385 (54.0)	466 (52.1)	1.07 (0.88–1.31)	0.98 (0.76–1.25)
Female infertility	145 (20.3)	149 (16.7)	1.27 (0.99–1.64)	1.22 (0.93–1.61)
Knowledge				
DOTS duration	713 (90.9)	894 (89.0)	1.22 (0.88–1.70)	0.86 (0.56–1.31)
Curable	648 (90.9)	796 (89.0)	**1.96** (2.43–2.69)*	**1.67** (1.17–2.39)*
Contagious	376 (52.7)	477 (53.4)	0.98 (0.81–1.20)	1.05 (0.83–1.32)
Vaccine	267 (37.4)	282 (31.5)	**1.29** (1.05–1.59)*	**1.35** (1.07–1.70)*
Advertisement				
Yes	642 (90.0)	781 (87.4)	1.30 (0.95–1.79)	1.18 (0.83–1.68)
Time to DOTS centre (minutes)				
<30	470 (65.9)	587 (65.7)	0.97 (0.79–1.20)	Ref
>30	205 (28.8)	264 (29.5)	1.02 (0.83–1.26)	**1.72** (1.08–2.74)*
Waiting time at DOTS center (minutes)				
<30	538 (75.5)	687 (76.8)	0.93 (0.74–1.17)	Ref
>30	151 (21.2)	175 (19.6)	1.07 (0.85–1.35)	0.98 (0.75–1.27)
Provider explained				
DOTS duration	683 (95.8)	823 (92.1)	**1.96** (1.26–3.04)*	**1.88** (1.12–3.15)*

*Statistically significant.
